# Antioxidant effects of pineapple vinegar in reversing of paracetamol-induced liver damage in mice

**DOI:** 10.1186/s13020-015-0030-4

**Published:** 2015-02-13

**Authors:** Nurul Elyani Mohamad, Swee Keong Yeap, Kian Lam Lim, Hamidah Mohd Yusof, Boon Kee Beh, Sheau Wei Tan, Wan Yong Ho, Shaiful Adzni Sharifuddin, Anisah Jamaluddin, Kamariah Long, Nik Mohd Afizan Nik Abd Rahman, Noorjahan Banu Alitheen

**Affiliations:** Department of Cell and Molecular Biology, Faculty of Biotechnology and Biomolecular Science, Universiti Putra Malaysia, Serdang, Selangor 43400 Malaysia; Institute of Bioscience, Universiti Putra Malaysia, Serdang, Selangor 43400 Malaysia; Faculty of Medicine and Health Sciences, Universiti Tunku Abdul Rahman, Sungai Long Campus, Jalan Sungai Long, Bandar Sungai Long, Cheras, Kajang, 43000 Selangor Malaysia; School of Biomedical Sciences, The University of Nottingham Malaysia Campus, Jalan Broga, 43500, Semenyih, Selangor 43400 Malaysia; Biotechnology Research Centre, Malaysian Agricultural Research and Development Institute (MARDI), Serdang, Selangor 43400 Malaysia

## Abstract

**Background:**

Pineapple (*Ananas comosus*) was demonstrated to be hepatoprotective. This study aims to investigate the reversing effects of pineapple vinegar on paracetamol-induced liver damage in murine model.

**Methods:**

Pineapple juice was fermented *via* anaerobic and aerobic fermentation to produce pineapple vinegar. Male BALB/c mice (n = 70) were separated into 7 treatment groups (n = 10). Pineapple vinegar (0.08 and 2 mL/kg BW) and synthetic vinegar were used to treat paracetamol-induced liver damage in mice. The hepatoprotective effects were determined by serum biochemistry profiles (aspartate aminotransferase (AST), alanine aminotransferase (ALT), alkaline phosphatase (ALP), and triglyceride (TG)), liver antioxidant levels (ferric-reducing ability plasma (FRAP), superoxide dismutase (SOD), malondialdehyde (MDA), nitric oxide (NO), and reduced glutathione assays (GSH)) and histopathological examination with hematoxylin and eosin (H&E) staining. The effects were further evaluated by the expression levels of iNOS, NF-κB, and cytochrome P450 2E1 by quantitative real-time PCR and Western blot analyses. Vinegar samples were also tested for *in vitro* antioxidant (FRAP, 2,2-diphenyl-2-picrylhydrazyl (DPPH), and total phenolic content (TPC)). Soluble phenolic acid contents in the samples were identified by HPLC.

**Results:**

Pineapple vinegar contained 169.67 ± 0.05 μg GAE/mL of TPC, with 862.61 ± 4.38 μg/mL gallic acid as the main component. Oral administration of pineapple vinegar at 2 mL/kg BW reduced serum enzyme biomarker levels, including AST (*P* = 0.008), ALT (*P* = 0.006), ALP (*P*_*=*_ 0.002), and TG (*P =* 0.006) after 7 days of paracetamol treatment. Liver antioxidant levels such as hepatic glutathione (*P* = 0.003), SOD (*P* < 0.001), lipid peroxidation (*P* = 0.002) and FRAP (*P <*0.001) were restored after the treatment. Pineapple vinegar reduced the expressions of iNOS (*P* = 0.003) and NF-kB (*P* = 0.003) and the level of NO (*P* = 0.003) significantly. Pineapple vinegar also downregulated liver cytochrome P450 protein expression.

**Conclusions:**

Oral administration of pineapple vinegar at 0.08 and 2 mL/kg BW reduced serum enzyme biomarker levels, restored liver antioxidant levels, reduced inflammatory factor expressions, and down regulated liver cytochrome P450 protein expression in paracetamol-induced liver damage in mice.

## Background

The functions of the liver, such as fatty acid metabolism [1], protein synthesis [2], and detoxification [3], would be impaired by excessive intake of ethanol, carbon tetrachloride (CCl_4_), and iron, causing accumulation of free radicals in the body [4]. Over-consumption of paracetamol causes hepatic necrosis and inflammation through activation of cytochrome P450 by N-acetyl-p-benzoquinoneimine (NAPQI) in paracetamol [5,6]. During the process, reactive oxygen species (ROS) are produced excessively [7]. Fruits are rich in antioxidant compounds that may help protect the body against ROS-mediated damage [8]. Vinegar produced from fruits enhanced the reduced glutathione (GSH) antioxidant system and scavenged the radical activities, thereby protecting the liver cells from damage [9].

Vinegar produced from carbohydrate sources, such as fruits and grains, contains not only acetic acid, but also other bioactive compounds such as polyphenolics, volatile compounds, and organic acids [10]. Production of vinegar involves alcoholic and acetic fermentations. Yeast converts sugar into alcohol during alcoholic fermentation, which is then transformed into acetic acid by *Acetobacter* bacteria during acetic fermentation [10]. Many studies have evaluated the functionality and pharmacological effects of vinegar in treating and preventing certain diseases such as hypercholesterolemia [11], hyperglycemia [12], hypertension [13], and cancer [14]. Vinegar also possessed an anti-thrombotic effect [15] and antimicrobial activity [16]. Vinegar produced from *Hovenia dulcis* peduncles shows a hepatoprotective effect against alcohol-induced liver damage in mice [17].

Pineapple (*Ananas comosus*) is one of the carbohydrate sources for vinegar production. It is commonly grown in tropical and subtropical countries, including Hawaii, India, China, Kenya, South Africa, Malaysia, Philippines, and Thailand [18]. Pineapple contains phenolic compounds, vitamins, and several proteinases such as bromelain, comosain, and ananain, which are antioxidants [19]. According to the literature, pineapple protects the liver intoxicated by paracetamol-induced damages in rats [20,21]. However, there have been no scientific reports on the effects of pineapple vinegar on paracetamol-induced hepatotoxicity.

This study aims to investigate the hepatoprotective effects of pineapple vinegar on paracetamol-induced liver damage in male BALB/c mice.

## Methods

### Chemicals and samples

Folin–Ciocalteu reagent, 2,2-diphenyl-2-picrylhydrazyl (DPPH), 2,4,6-tripyridyl-*s*-triazine (TPTZ), FeCl_3_.6H_2_0, gallic acid, and trolox were purchased from Sigma (USA). Silybin was purchased from BiO-LiFE (Malaysia). *Saccharomyces cerevisiae 7013 INRA* and *Acetobacter acetii var. Europeans* used in the production of vinegar were obtained from the culture collection center of the Malaysian Agriculture and Research Development Institute (MARDI), Malaysia.

A vinegar sample was made from pineapple fruit obtained from a local market (Pasar Borong Selangor, Selangor). Briefly, the pineapple fruits were sliced and blended to produce the juice. The juice was inoculated with *S. cerevisiae 7013 INRA* for 7–10 days under anaerobic fermentation at 28–30°C to produce alcohol, and then further inoculated with *Acetobacter acetii vat Europeans* to allow aerobic fermentation to occur at 28–30°C. After 4 weeks of incubation, 6–8% acetic acid was produced and the solution was transferred to a storage tank for 1 month for the maturation process. Finally, the vinegar product was filtered, placed in a glass bottle (Schott, Germany), and stored at 4°C. Samples for this study were freshly prepared daily by dilution of the vinegar product with distilled water.

### Animals

Male BALB/c mice (4–5 weeks of age) with an average weight of 20–22 g were obtained from the Animal House of the Faculty of Veterinary Sciences, Universiti Putra Malaysia. The mice were placed in plastic cages at room temperature (21–23°C) with a 12-h/12-h dark/light cycle and relative humidity of approximately 60%. They received a standard pellet diet and distilled water *ad libitum* and were acclimatized for 7 days prior to the experiment. This study was approved by the Animal Care and Use Committee, Universiti Putra Malaysia (UPM/FPV/PS/3.2.1.551/AUP-R168), and conducted according to the guidelines from the committee.

### Experimental design

The mice were arbitrarily divided into seven groups with ten mice each. The treatment was performed according to a previous study with slight modifications [22]. The high-dose vinegar group was designed according to a previous *in vivo* study with a vinegar sample [23], while the low-dose vinegar group was modified to reduce the dose and make it equivalent to the concentration of one tablespoon of vinegar in a 250 mL glass of water. All groups, except for the normal control group (group 1), were treated with paracetamol at 250-mg/kg body weight (BW) to induce liver damage in mice *via* oral gavage for 7 days. After 7 days, all mice were subjected to oral treatments once daily for 14 days *via* oral gavage as follows:

Group 1: normal control group without paracetamol induction receiving distilled water only;

Group 2: untreated paracetamol control group receiving distilled water only;

Group 3: positive control group receiving 50-mg/kg BW of silybin;

Group 4: acetic acid control group receiving 2-mL/kg BW of synthetic vinegar;

Group 5: acetic acid control group receiving 0.08-mL/kg BW of synthetic vinegar;

Group 6: treatment group receiving 2 mL/kg-BW of pineapple vinegar; and

Group 7: treatment group receiving 0.08-mL/kg BW of pineapple vinegar.

At the end of the experimental period, all mice were anesthetized with isoflurane, and euthanized by cervical dislocation. Liver and serum were collected and subjected to the following assays.

### Serum biomarker assays

Sera were collected for quantification of the following enzyme markers: aspartate aminotransferase (AST), alanine aminotransferase (ALT), alkaline phosphatase (ALP), and triglyceride (TG). The assays were performed in a biochemical analyzer (Hitachi 902 Automatic Analyzer; Hitachi, Japan) with adapted reagents from Roche (Germany).

### Liver antioxidant levels

The liver was excised from each mouse, washed with ice-cold phosphate buffer (137-mM NaCl, 2.7-mM KCl, 10-mM Na_2_HPO_4_, 2-mM KH_2_PO_4_, pH 7.4), and weighed before being divided into several parts. One part of the liver was mashed using a 0.2-μm cell strainer (SPL Life Sciences, China) and syringe rubber plunger in cold phosphate-buffered saline (PBS) to obtain a liver homogenate. The liver homogenate was used to determine the levels of ferric-reducing ability plasma (FRAP), superoxide dismutase (SOD), malondialdehyde (MDA), and nitric oxide (NO) according to previously described methods [24], while the GSH levels were determined with a Glutathione Assay Kit (Sigma-Aldrich, USA).

#### FRAP assay

A master solution was prepared by mixing 30 mL of 300-mM acetate buffer with 3 mL of 10-mM TPTZ solution and 3 mL of 20-mM FeCl_3_⋅6H_2_O solution in 40-mM HCl. The solution was kept in the dark at 37°C. For the assay, 80 μL of liver homogenate and 150 μL of master solution were added to a 96 well-plate and mixed thoroughly. After 10 min of incubation, the absorbances were measured at 593 nm in an ELISA Plate Reader (Bio-Tek Instruments, USA) and the activity was calculated from a standard FeSO_4_ calibration curve.

#### SOD assay

A master solution was prepared by adding 0.1-mol/L phosphate buffer, 0.15-mg/mL sodium cyanide in 0.1-mol/L ethylenediaminetetraacetic acid (EDTA), 1.5-mmol/L nitroblue tetrazolium, and 0.12-mmol/L riboflavin. In brief, 100 μL of a serial dilution of liver homogenates were pipetted into a 96-well plate and mixed well with 200 μL of master solution. The absorbances were then measured at 560 nm using ELISA Plate Reader (Bio-Tek Instruments, USA) and the activity was expressed as units SOD/mg protein.

#### MDA assay

Each liver homogenate (200 μL) was diluted with 800 μL of PBS and mixed with 25 μL of 8.8 mg/mL butyhydroxytoluene and 500 μL of 50% trichloroacetic acid. The mixture was vortexed, incubated for 2 h on ice, and centrifuged (MX-160 Tomy, Japan) at 2000 × *g* for 15 min. The supernatant (1 mL) was transferred into a new tube and mixed with 75 μL of 0.1-M EDTA and 250 μL of 0.05-M 2-thiobarbituric acid. The mixture was boiled for 15 min and allowed to cool to room temperature before the absorbances were measured at 532 and 600 nm in an ELISA Plate Reader (Bio-Tek Instruments, USA).

#### NO assay

NO activity was determined using a Griess reagent kit (Invitrogen, USA) according to the manufacturer’s protocol. Griess reagent (20 μL) was mixed with 150 μL of liver homogenate and 130 μL of distilled water in a 96 well-plate and incubated for 30 min at room temperature. The absorbances were measured at 540 nm in an ELISA Plate Reader (Bio-Tek Instruments, USA).

#### GSH activity assay

The GSH level was determined using a Glutathione Assay Kit (Sigma-Aldrich, USA). Briefly, 10 mL of liver homogenate and 150 μL of working solution (1.5 mg/mL DTNB, 6 U/mL glutathione reductase, and 1× assay buffer) were added to a 96 well-plate and mixed thoroughly. The plate was incubated for 5 min before 50 mL of NADPH solution (0.16 mg/mL) was added to each well. The absorbances were measured at 412 nm in an ELISA Plate Reader (Bio-Tek Instruments, USA) at 1-min intervals for 5 min.

### RNA isolation and quantitative real-time PCR gene expression analysis

Livers were stored in RNA*later* solution (Life Technologies, USA) to preserve the RNA. The RNA was then extracted using an RNeasy Mini Kit (Qiagen, Germany). Total RNA (1 μg) was reverse-transcribed to first-strand cDNA using iScript™ Reverse Transcription Supermix for RT-qPCR (Bio-Rad, USA) according to the manufacturer’s protocols. Quantitative real-time PCR was performed with iTaq™ Universal SYBR® Green Supermix (Bio-Rad, USA). The following primers were used: nuclear factor kappa-light-chain-enhancer of activated B cells (NF-κB): forward 5′-CATTCTGACCTTGCCTATCT-3′ and reverse 3′-CTGCTGTTCTGTCCATTCT-5′; inducible nitric oxide synthase (iNOS): forward 5′-GCACCGAGATTGGAGTTC-3′ and reverse 3′-GAGCACAGCCACATTGAT-5′; beta-actin (ACTB): forward 5′-TTCCAGCCTTCCTTCTTG-3′ and reverse 3′-GGAGCCAGAGCAGTAATC-5′; hypoxanthine phosphoribosyl transferase (HPRT): forward 5′-CGTGATTAGCGATGATGAAC-3′ and reverse 3′-AATGTAATCCAGCAGGTCAG-5′; and glyceraldehyde 3-phosphate dehydrogenase (GAPDH): forward 5′-GAAGGTGGTGAAGCAGGCATC-3′ and reverse 3′-GAAGGTGGAAGAGTGGGAGTT-5′. The quantities of the target genes and the housekeeping genes ACTB, HPRT, and GAPDH were calculated according to a standard curve and the expressions of NF-κB and iNOS were measured using CFX Manager Software (Bio-Rad, USA). The expression levels in all samples were compared with those in the untreated control group, and the levels of the different mRNAs in the untreated control group were designated as 1. All results were expressed as fold changes and measured in triplicate. Non-template controls were used to confirm specificity.

### Western blotting

P450 protein expression levels were determined by Western blotting [25] with beta-actin (ab8227; Abcam, USA) as a housekeeping control. Briefly, total protein was extracted from 30 mg of fresh liver tissues using RIPA buffer (50-mM Tris pH 8, 150-mM NaCl, 1% Triton X-100, 0.1% sodium deoxycholate, 0.1% SDS) supplemented with a phosphatase inhibitor cocktail (Roche, Canada). Next, aliquots containing 100 μg of protein were separated by 10% sodium dodecyl sulfate-polyacrylamide gel electrophoresis and transferred to a nitrocellulose membrane (Roche, Canada) using a Pierce Fast Semi-Dry Blotter (Pierce, USA). The membrane was blocked with 1% skimmed milk for 2 h, washed with TBST buffer (1.5-M NaCl, 0.5-M Tris, pH 7.5) three times, and incubated with an anti-cytochrome P450 2E1 antibody (ab28146; Abcam, USA). Next, the membrane was incubated with a secondary antibody conjugated with horseradish peroxidase (Abcam, USA). The chemiluminescence signals (Super Signal West Pico; Pierce, USA) were developed using a Chemi Doc UVP machine (UVP, USA). The density results were analyzed using Vision Work LS Analysis software (UVP, USA).

### Histopathology

One part of the fresh liver tissues was placed in plastic cassettes and immersed in neutral-buffered formalin for 24 h [26]. The fixed tissues were embedded in paraffin, sectioned, deparaffinized, rehydrated using standard techniques, and stained with hematoxylin and eosin. The stained liver sections were observed under a Nikon Eclipse 90i microscope (Nikon, USA) using bright-field optics at 40× magnification.

### Determination of total phenolic contents and soluble phenolic acid compounds

The total phenolic contents of vinegar samples were determined by the Folin–Ciocalteu assay and the results were expressed in milligrams of gallic acid [27]. Briefly, samples were incubated with Folin–Ciocalteu reagent (Sigma, USA) for 3–8 min. The mixture was then added with 0.8 mL of 7.8% sodium carbonate (Sigma, USA) solution and incubated at room temperature for 2 h. The absorbances were measured at 765 nm in an ELISA Plate Reader (Bio-Tek Instruments, USA).

Free phenolic acids in vinegar samples were quantified using an HPLC Alliance Separation Module (Waters, USA) equipped with a diode array detector as described in [28] with slight modifications. Briefly, undiluted 10-μL samples were injected into a reverse-phase analytical column (150 mm × 4.6 mm × Bridge C18, 3.5 μm; Waters, USA) at a controlled temperature of 25°C for separation. The mobile phase was 0.1% formic acid and methanol with a flow rate of 0.7 mL/min. The data obtained were analyzed against corresponding standards (gallic acid, protocatechuic acid, β-hydroxybenzoic acid, vanillic acid, caffeic acid, syringic acid, sinapic acid, and benzoic acid).

### *In vitro* evaluation of antioxidant activities of vinegar samples

#### DPPH assay

The free-radical-scavenging activities of the vinegar samples were measured by the DPPH assay with trolox as the standard [29]. Briefly, 50 μL of pineapple vinegar and synthetic vinegar were added to 250 μL of DPPH working solution and incubated in the dark for 30 min. The absorbances were measured in an ELISA Plate Reader (Bio-Tek Instruments, USA).

#### FRAP assay

The FRAP assay was performed according to Thaipong *et al.* [29] with slight modifications. A working solution was prepared by adding 4 mL of TPTZ and 4 mL of FeCl_3_.6H_2_0 to 40 mL of acetate buffer. The solution was kept at 37°C in the dark. For the assay, 20-μL aliquots of samples were added to 150 μL of FRAP working solution and incubated for 10 min. The absorbances were measured at 593 nm in an ELISA Plate Reader (Bio-Tek Instruments, USA). The results were calculated from a standard FeSO_4_ calibration curve and expressed in μM Fe^2+^.

### Statistical analysis

All assays were repeated in three independent experiments. Means ± standard deviations (SD) were compared for each group by one-way analysis of variance (ANOVA) and Duncan’s multiple range test using SPSS 16.0 statistical software (IBM, USA). *P* values <0.05 were considered statistically significant. Dose-dependent manner was visually determined by observing the trend of the data.

## Results

### Serum biochemistry

The effects of silybin, synthetic vinegar, and pineapple vinegar on serum biomarkers in paracetamol-treated mice are summarized in Table 1. The levels of ALT, ALP, AST, and TG in the paracetamol-untreated control group were elevated by 2, 1.2, 2.5, and 1.5-fold [*P*_*ALT*_ = 0.033, *P*_*ALP*_ = 0.023, *P*_*AST*_ = 0.020, *P*_*TG*_ = 0.022], respectively, compared with the normal control group. The elevations of these marker enzymes indicated hepatic injury and loss of structural integrity in liver cells. Treatments with silybin [*P*_*ALT*_ = 0.003, *P*_*ALP*_ = 0.003, *P*_*AST*_ = 0.002, *P*_*TG*_ = 0.010*]* synthetic vinegar [*P*_*ALT*_ = 0.010, *P*_*ALP*_ = 0.003, *P*_*AST*_ = 0.002, *P*_*TG*_ = 0.002.], and pineapple vinegar [*P*_*ALT*_ = 0.002, *P*_*ALP*_ = 0.003, *P*_*AST*_ = 0.002, *P*_*TG*_ = 0.006] significantly restored the concentrations of these enzymes to almost their normal levels. The treatments were dose-dependent (Table 1), the higher dosages of the vinegar samples were able to reduce the serum marker enzymes more effectively than the lower dosages.

**Table 1:** **Serum biochemical parameters of different experimental groups on paracetamol (PCM) induced hepatotoxicity in mice**

**Group (n = 10)**	**ALT (U/L)**	**ALP (U/L)**	**AST (U/L)**	**Triglyceride (mmol/L)**
1	61.23 ± 5.57*	85.67 ± 2.32*	145.20 ± 15.15*	2.33 ± 0.64*
2	123.94 ± 7.25	104.44 ± 2.31	368.76 ± 9.83	3.44 ± 0.56
3	72.44 ± 8.23*	81.75 ± 1.51*	250.46 ± 11.14*	2.11 ± 0.24*
4	45.44 ± 2.63*	85.83 ± 2.55*	172.64 ± 10.58*	2.53 ± 1.11*
5	76.53 ± 4.15*	91.83 ± 1.25*	235.96 ± 13.19*	2.63 ± 1.06*
6	45.26 ± 7.59*	75.75 ± 1.77*	184.03 ± 28.88*	1.99 ± 0.46*
7	74.31 ± 7.35*	85.75 ± 2.88*	239.33 ± 28.24*	2.83 ± 0.69*

Values are expressed as mean ± SD where *indicates that the values are significantly difference from paracetamol control group, *P <* 0.05.

Group1: normal mice; group 2: paracetamol control (250 mg/kg PCM + PBS); group 3: positive control (250 mg/kg PCM + 50 mg/kg silybin); group 4: acetic acid control (250 mg/kg PCM + 2 mL/kg synthetic vinegar; group 5: acetic acid control (250 mg/kg PCM + 0.08 mL/kg synthetic vinegar; group 6: 250 mg/kg PCM + 2 mL/kg pineapple vinegar; group 7: 250 mg/kg PCM + 0.08 mL/kg pineapple vinegar.

### Liver antioxidant and NO determinations

The antioxidant activities in paracetamol-intoxicated mice were evaluated by several antioxidant assays, comprising GSH, SOD, FRAP, MDA, and NO assays (Table 2). The increases in antioxidant strength (GSH, SOD, and FRAP) led to depletion of the MDA and NO levels. Both high concentration pineapple vinegar and synthetic vinegar improved the GSH content [*P*_*PH*_ = 0.003, *P*_*SH*_ = 0.013], SOD [*P*_*PH*_ <0.001, *P*_*SH*_ =0.002.], and FRAP [*P*_*PH*_ <0.001, *P*_*SH*_ =0.003], and reduced NO [*P*_*PH*_ = 0.002, *P*_*SH*_ =0.033] and MDA [*P*_*PH*_ = 0.002, *P*_*SH*_ = 0.030] significantly, and the effects were dose-dependent. However, due to the differences in phenolic contents between the samples, high-dose pineapple vinegar provided better recovery in abolishing the effects of paracetamol-induced liver damage compared with the other treatments.

**Table 2:** **Liver antioxidant and NO determination of different experimental groups on paracetamol (PCM) induced hepatotoxicity in mice**

**Group (n = 10)**	**GSH (nm GSH/mg protein)**	**SOD (U/mg protein)**	**FRAP (μM Fe(II)/mg protein)**	**MDA (nM MDA/mg protein)**	**NO (μM/mg protein)**
**1**	4.16 ± 0.04*	122.18 ± 1.09*	21.13 ± 1.99*	1.53 ± 0.28*	20.71 ± 2.67*
**2**	2.55 ± 0.03	41.50 ± 1.38	12.77 ± 1.68	5.14 ± 0.10	44.74 ± 1.05
**3**	4.63 ± 0.03*	145.47 ± 2.87*	20.73 ± 1.78*	1.63 ± 0.21*	27.61 ± 3.08*
**4**	4.78 ± 0.01*	125.33 ± 1.65*	20.77 ± 5.85*	1.65 ± 0.24*	32.45 ± 3.21*
**5**	4.26 ± 0.04*	84.99 ± 2.53*	14.78 ± 3.30	2.50 ± 0.57*	36.68 ± 6.20
**6**	7.57 ± 0.12*	203.53 ± 3.64*	26.02 ± 2.10*	1.51 ± 0.01*	19.74 ± 0.87*
**7**	5.54 ± 0.03*	139.55 ± 1.52*	20.87 ± 2.65*	1.56 ± 0.09*	31.66 ± 4.87*

Values are expressed as mean ± SD where * indicates that the values are significantly difference from paracetamol control group, *P <* 0.05.

Group1: normal mice; group 2: paracetamol control (250 mg/kg PCM + PBS); group 3: positive control (250 mg/kg PCM + 50 mg/kg silybin); group 4: acetic acid control (250 mg/kg PCM + 2 mL/kg synthetic vinegar; group 5: acetic acid control (250 mg/kg PCM + 0.08 mL/kg synthetic vinegar; group 6: 250 mg/kg PCM + 2 mL/kg pineapple vinegar; group 7: 250 mg/kg PCM + 0.08 mL/kg pineapple vinegar.

### Liver histopathology

**Figure 1:**
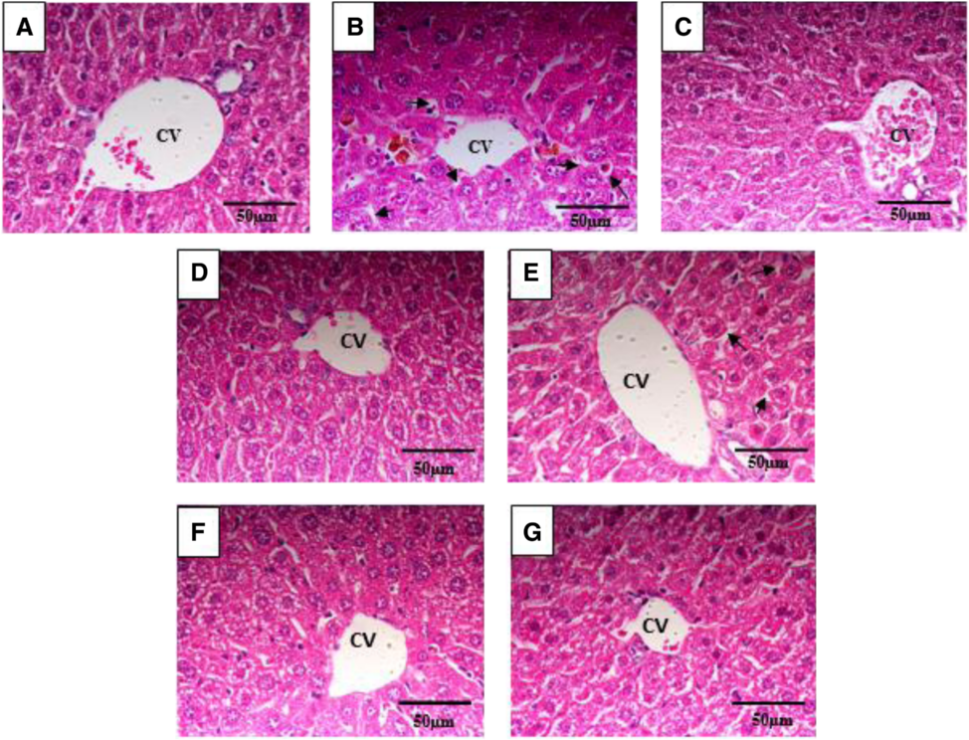
**Histopathological change in liver (H&E stanining).** Photomicrograph of liver section (40 x) for **(A)** normal hepatocytes; **(B)** untreated group (negative control) after administration of 250 mg/kg paracetamol showing microvesicular steatosis (arrow) in the liver parenchyma; **(C)** paracetamol group after administration of 50 mg/kg silybin showing minimal microvesicular steatosis; **(D)** paracetamol group after administration of 2 mL/kg of synthetic vinegar showing the development of microvesicular steatosis (arrow) in the liver parenchyma; **(E)** paracetamol group after administration of 0.08 mL/kg of synthetic vinegar with recovery effect of microvesicular steatosis; **(F)** paracetamol group after administration of 2 mL/kg of pineapple vinegar showing the lesser development of microvesicular steatosis (arrow) in the liver parenchyma; **(G)** paracetamol group after administration of 0.08 mL/kg of pineapple vinegar with recovery effect of fatty changes. CV is central vain; arrow indicates microvesicular steatosis.

Histopathological assessments using liver sections were performed for all experimental groups by hematoxylin and eosin staining (Figure 1). In the paracetamol-induced hepatotoxicity group, microvesicular steatosis, and accumulation of fat in the liver were observed in the paracetamol-induced hepatotoxicity group. Treatments with silybin and high concentrations of synthetic vinegar and pineapple vinegar caused mild fatty changes in the hepatic parenchyma. The nucleus and cytoplasm of cells were well-shaped and no changes were observed in their structures (Figure 1A). In contrast, changes in the liver structure such as microvesicular steatosis (arrow), regeneration in the nucleus with clumping, increase in nuclear size, and ballooning were observed in the untreated control group given paracetamol (Figure 1B). Figures 1C,D,E,F, and G show liver sections from the groups treated with silybin, high-dose synthetic vinegar, low-dose synthetic vinegar, high-dose pineapple vinegar, and low-dose pineapple vinegar, respectively. The recovery in the synthetic vinegar groups was much lower than that in the pineapple vinegar groups, with moderate recovery in the high-dose synthetic vinegar group. Normalization of hepatocytes into normal structures with significant reductions in microvesicular steatosis, ballooning, and hepatocyte necrosis were observed for both concentrations of pineapple vinegar.

### Quantitative PCR gene expression analyses for iNOS and NF-κB in the liver

**Figure 2:**
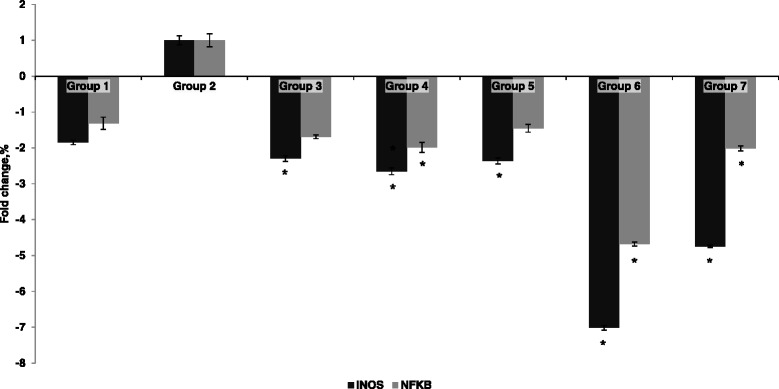
**Liver gene expressions.** Downregulation of iNOS and NF-κB in different treatment groups of paracetamol induced liver damage in mice. Relative expression of iNOS and NF-κB genes by different treatment groups were normalized to the expression of the paracetamol control group. Group 1: normal mice; group 2: paracetamol control (250 mg/kg PCM + PBS); group 3: positive control (250 mg/kg PCM + 50 mg/kg silybin); group 4: acetic acid control (250 mg/kg PCM + 2 mL/kg synthetic vinegar; group 5: acetic acid control (250 mg/kg PCM + 0.08 mL/kg synthetic vinegar; group 6: 250 mg/kg PCM + pineapple vinegar (2 mL/kg); group 7: 250 mg/kg PCM + pineapple vinegar (0.08 mL/kg). Values were significant difference compared with paracetamol control group, *P <* 0.05. Values are expressed as mean ± SD where * indicates that the values are significantly difference from paracetamol control group, *P <* 0.05.

The expression levels of the iNOS and NF-κB genes in the liver were analyzed by quantitative PCR (Figure 2). Oral administration of both concentrations of pineapple vinegar significantly decreased the iNOS [*P*_*PH*_ = 0.003, *P*_*PL*_ = 0.003] and NF-κB [*P*_*PH*_ = 0.003, *P*_*PL*_ = 0.003] expression levels compared with the paracetamol control group, and the greatest downregulation of both genes was observed in the high-dose pineapple vinegar group.

### Western blot analysis of cytochrome P450 levels in the liver

**Figure 3:**
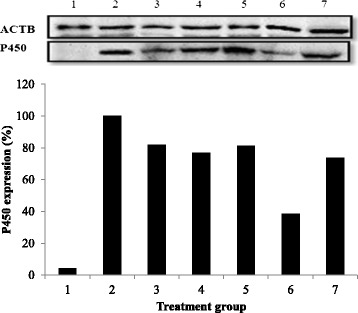
**Liver P450 expressions.** Protein samples were subjected to electro-transfer to a nitrocellulose membrane, incubated with primary antibodies against ACTB and anti-rabbit secondary antibodies conjugated with HRP. Lane 1: normal; lane 2: untreated; lane 3: silybin; lane 4: synthetic vinegar (2 mL/kg); lane 5: synthetic vinegar (0.08 mL/kg); lane 6: pineapple vinegar (2 mL/kg); lane 7: pineapple vinegar (0.08 mL/kg).

Figure 3 shows the cytochrome P450 2E1 protein expression levels in the liver tissues of the different experimental groups. The P450 protein expression level was significantly decreased by 62% in the high-dose pineapple vinegar group compared with the paracetamol control group. Moderate decreases were noted in the other treatment groups (silybin, high-dose synthetic vinegar, low-dose synthetic vinegar, and low-dose pineapple vinegar) with no significant differences between the groups.

### Total phenolic and soluble phenolic acid contents in pineapple vinegar

From the calorimetric assay results, the total phenolic content in pineapple vinegar was 169.67 ± 0.05-μg GAE/mL, while synthetic vinegar had no phenolic content. HPLC was carried out to confirm these results and further investigate the phenolic acid derivatives in both samples. From the HPLC results, gallic acid (862.61 ± 4.38 μg/mL) was the main component of the phenolic compounds in pineapple vinegar followed by caffeic (218.91 ± 3.24 μg/mL) and benzoic acid (177.90 ± 14.02 μg/mL), while no phenolic compounds were detected in synthetic vinegar. The total phenolic contents and phenolic acid derivatives are shown in Table 3.

**Table 3:** **Total phenolic, soluble phenolic acids content, DPPH and FRAP of pineapple vinegar**

	**Pineapple vinegar**	**Synthetic vinegar**
Total phenolic content (μg GAE/mL)	169.67 ± 0.05	ND
Phenolic acid derivatives (HPLC):		
Gallic acid (μg/mL)	862.61 ± 4.38	ND
Caffeic acid (μg/mL)	218.91 ± 3.24	ND
Benzoic acid (μg/mL)	177.90 ± 14.02	ND
Sinapic acid (μg/mL)	154.28 ± 4.09	ND
Vanillic acid (μg/mL)	117.35 ± 3.99	ND
β-hydroxybenzoic (μg/mL)	83.99 ± 1.15	ND
Protocatechuic acid (μg/mL)	78.75 ± 1.70	ND
Syringic acid (μg/mL)	55.46 ± 9.51	ND
DPPH (%)	69.28 ± 0.18	ND
FRAP (μg TE/mL)	357.72 ± 0.07	ND

ND indicates not detected.

### *In vitro* evaluation of antioxidant activities of vinegar samples

The antioxidant strength of pineapple vinegar was tested by FRAP and DPPH radical-scavenging activity. The inhibitory concentration (IC_50_) values for the radical-scavenging activities of pineapple vinegar in this study were 69.28 ± 0.18% and 357.72 ± 0.07-μg TE/mL for the FRAP assay. No values for FRAP and no IC_50_ values for radical-scavenging activities were found in synthetic vinegar.

## Discussion

Overdose of paracetamol causes liver inflammation, which results in increased TG levels due to impaired fat metabolism and fatty changes [30]. Change or loss of the structural integrity of hepatocytes caused residing enzymes, such as ALT, ALP, and AST, to leak out and increase their concentrations in the blood [31]. Decreases in TG levels and these enzymes showed hepatoprotective effects and restored the hepatocyte structure [32]. Acetic acid improved fatty acid oxidation in the liver [33,34], thereby lowering TG levels in blood and reducing microvesicular steatosis development in liver tissues. Additional mechanisms through active constituents such as flavonoids and silybin restored these enzymes to protect liver cells [17] by preventing leakage of the intracellular enzymes through repair and stabilization of the hepatocyte membrane [35]. Reduced microvesicular steatosis and ballooning were observed in liver tissues in all treatment groups. The present results demonstrated that the recovery in mice treated with pineapple vinegar was better than that in the mice treated with synthetic vinegar due to its antioxidant strength.

Phenolic compounds are secondary metabolites in plants and plant products that contribute to their antioxidant activities [36]. Improved GSH levels in the liver reversed the effects of paracetamol-induced liver injury through conjugation between GSH and NAPQI [37]. The total reducing and antioxidant capacities of the vinegar samples decreased NO and lipid peroxidation, which resulted in decreased MDA levels in the liver [38]. From the antioxidant results shown in Table 2, both pineapple vinegar and synthetic vinegar increased the antioxidant levels in liver homogenates, although pineapple vinegar acted as a more effective hepatoprotectant than synthetic vinegar. The acetic acid contents in the samples may also contribute to the antioxidant activities in the liver and restoration of liver marker enzymes. To date, there have been no reports on the possible mechanisms of acetic acid in treating hepatotoxicity. However, because of the capability of acetic acid to reduce the fatty changes in hepatocytes, it might assist in the restoration and preservation of the antioxidant potential of liver cells [33].

Western blot and quantitative PCR analyses were performed to assess gene and protein expressions. NF-κB, one of the inflammatory reactants, is a transcriptional factor that is activated in most cell types as a response to foreign pathogens or general stress insults [39]. Activation of NF-κB upregulates the expressions of inflammatory genes such as iNOS [40]. The present results demonstrated that a high concentration of pineapple vinegar downregulated the expression of iNOS. This finding was supported by the reduction in the NO levels, indicating that the high dose of pineapple vinegar significantly reduced paracetamol-induced liver inflammation.

Any sample or compound that induces cytochrome P450 2E1 can induce hepatotoxicity [41,42], including paracetamol [43], while improved levels of GSH and antioxidants reduce the levels of cytochrome P450 2E1 [44]. Under oxidative stress, NO induced detoxification by increasing the levels of SOD and GSH [45], and restored balance to the liver cells by conjugation with the reactive metabolite [46]. As shown in Figure 3, the significant down-regulation of P450 observed in the mice treated with the high dose of pineapple vinegar might be attributed to the increased GSH and antioxidant levels in the samples evaluated by western blotting.

Pineapple is a good source of antioxidants [47]*.* Antioxidant strength is measured by both phenolic and non-phenolic compounds, as they reverse free radical activities by decomposing peroxides and unpaired oxygen molecules, and adsorbing and neutralizing the free radicals to render them harmless to the body [48]. The fermentation process broke down undesirable compounds and increased the contents of some phenolic and non-phenolic compounds [49,50]. From both the *in vitro* and *in vivo* data in the present study, pineapple vinegar possessed high antioxidant activity compared with synthetic vinegar. As shown in Table 3, HPLC identified gallic acid as the active compound in pineapple vinegar, followed by caffeic, benzoic, sinapic, vanillic, β-hydroxybenzoic, protocatechuic, and syringic acids.

Gallic acid has been shown to suppress the activation of cytochrome P450 through its antioxidant and scavenging activities [51]. Furthermore, vanillic and syringic acids were found to suppress oxidative stress and reduce inflammation and fibrogenesis in CCl_4_-induced liver damage in BALB/c mice [52]. Sinapic acid significantly reduced lipid peroxidation and suppressed the activation of NF-κB in dimethylnitrosamine-induced fibrosis in rats [53]. Moreover, caffeic acid increased the GSH and catalase levels and reduced MDA lipid peroxidation in liver homogenates [54]. As shown in Table 3, all of these phenolic acid derivatives were major components of the phenolic compounds in pineapple vinegar. The hepatoprotective effects of pineapple vinegar may be contributed by these phenolic acid derivatives through their antioxidant strengths and scavenging activities, to restore the serum marker enzymes in the liver, repair the hepatocyte structure, and suppress the activation of cytochrome P450 through the downregulation of iNOS and NF-κB.

## Conclusions

Oral administration of pineapple vinegar at 0.08 and 2 mL/kg BW reduced serum enzyme biomarker levels, restored liver antioxidant levels, reduced inflammatory factor expressions, and down-regulated liver cytochrome P450 protein expression in paracetamol-induced liver damage in mice.

## Abbreviation

ACTB: β-actin
ALP: Alkaline phosphatase
ALT: Alanine aminotransferase
AST: Aspartate aminotransferase
CCL_4_: Carbon tetrachloride
DMN: dimethyl nitrosamine
DPPH: 2,2-diphenyl −2-picrylhydrazyl
FRAP: Ferric reduction ability plasma
GAPDH: Glyceraldehyde 3-phosphate dehydrogenase
GSH: Glutathione reductase
HPLC: High-performance liquid chromatography
HPRT: Hypoxanthine phosphoribosyltransferase
IC_50_: Inhibitory concentration
iNOS: Inducible nitric oxide synthase
MDA: Melonaldehyde
NAPQI: *N*-acetyl-*p*-benzoquinoneimine
NF-κB: Nuclear factor kappa-light-chain-enhancer of activated B cells
NO: Nitric oxide
qRT-PCR: Quantitative reverse transcriptase polymerase chain reaction
SDS-PAGE: Sodium dodecyl sulfate polyacrylamide gel electrophoresis
SOD: Superoxide dismutase
TG: Triglycride
TPTZ: 2,4,6-Tri(2-pyridyl)-s-triazine
